# Experimental Study on Active Cooling Systems Used for Thermal Management of High-Power Multichip Light-Emitting Diodes

**DOI:** 10.1155/2014/563805

**Published:** 2014-08-05

**Authors:** Mehmet Kaya

**Affiliations:** Department of Mechanical Engineering, Erzincan University, 24100 Erzincan, Turkey

## Abstract

The objective of this study was to develop suitable cooling systems for high-power multichip LEDs. To this end, three different active cooling systems were investigated to control the heat generated by the powering of high-power multichip LEDs in two different configurations (30 and 2 × 15 W). The following cooling systems were used in the study: an integrated multi-fin heat sink design with a fan, a cooling system with a thermoelectric cooler (TEC), and a heat pipe cooling device. According to the results, all three systems were observed to be sufficient for cooling high-power LEDs. Furthermore, it was observed that the integrated multifin heat sink design with a fan was the most efficient cooling system for a 30 W high-power multichip LED. The cooling system with a TEC and 46 W input power was the most efficient cooling system for 2 × 15 W high-power multichip LEDs.

## 1. Introduction

A light-emitting diode (LED) is actually a semiconductor diode. However, it differs from other normal diodes because it is manufactured for lighting applications. LEDs convert electrical energy to direct light [1, 2]. As the composition of different substances used to manufacture LEDs determines the colour of light emitted, LEDs can be manufactured to provide a wide spectrum of wavelengths, from infrared to ultraviolet. Lighting systems such as filament glow lamps and fluorescent tubes emit light by a heating or chemical process, whereas LEDs produce light through photons that are emitted from semiconductor junction areas [2]. Hence, LEDs have advantages such as longevity, higher efficiency, and smaller dimensions over other lighting systems.

In recent years, LEDs have been regarded as the most valuable light source because they have many advantages, such as energy consumption, durability, easy installation, flexible design, environmental compliance, higher light output with the same power consumption, rapid response, and longevity. LEDs have higher efficiency to reduce energy consumption. Because of reduction in the required energy, higher efficiency LEDs will reduce the using of fossil fuel that will provide a decrease in the environmental pollution and global warming caused by carbon dioxide and such gases [3–8]. Today, LEDs are replacing other light sources in many fields, such as indoor-outdoor and automotive lighting, screen backlighting, guideboards, signal systems, and panel screen lighting [4, 5]. The nature of LEDs is such that they convert approximately 20–30% of unused energy to light and convert the rest of the energy to heat [5, 9]. The heat adversely affects the light quality, efficiency, and longevity of LEDs. For example, a chip junction temperature above 150°C or a 10°C increase in an LED device increases the power used by the LED and also reduces light quality and LED life by half. Moreover, based on the physical interior structure of an LED, the LED's chip junction temperature must be kept under 120°C to prevent damage [4, 7, 10, 11]. For this reason, the heat generated by LEDs should be controlled via an efficient cooling system and extracted from LED device. The need for higher-level light sources requires the manufacturing of LEDs with higher energy levels. Compared to the heat generated by LEDs with lower energy levels, those with higher energy levels generate larger amounts of heat. Thus, such LEDs require a more efficient cooling system [4].

The literature suggests that there is no agreed-upon solution regarding the development of systems that are related to the efficient extraction of the heat generated by high-energy LEDs from LED devices themselves. Accordingly, studies are still being conducted on the development of effective and efficient cooling systems for high-energy LEDs. Therefore, the development of more efficient cooling systems for high-power LEDs remains a useful area of study [1, 4, 5, 7, 10, 12, 13]. Among these references, one of the well-known studies was performed by Lu et al. [1] to improve the thermal characteristics of high-power LED (light-emitting diode) package using a flat heat pipe. In that study obtained results indicated that the junction temperature of LED is about 52°C. To improve the heat dissipation of high-power light-emitting diodes having 6 × 3 W LEDs in two rows cooling system with thermoelectric cooler were used by Li et al. [4]. It was found that temperature of the substrate of LEDs reached 26°C without TEC, while it was only 9°C when the best refrigeration condition appears by using TEC. Another well-known study was done by Cheng et al. [5] to predict heat dissipation of high-power LED system and prediction of LED chip junction temperature by using finite element method. In that study it was found that, using a fan at the side wall of the heat sink channel to increase the convective heat transfer coefficient is an effective method to reduce the LED chip junction temperature. Moreover, Wang et al. [7] performed an experimental study to investigate the thermal performance of the vapor chamber which has been used for cooling system of 30 Watt high-power LEDs. They have found that plate works out hot-spot problem of 30 Watt high-power LEDs was successfully. Particularly, Li et al. [12] performed an experimental study on cooling of LED illumination package ranging from 30 W to 300 W using the loop heat pipe heat sink. They have indicated that measured thermal performance of loop heat pipe heat sink was superior to any conventional passive thermal management solutions in terms of heat sink weight.

Active and passive cooling systems are used to cool LEDs. Passive cooling systems are sufficient to cool low-power LEDs but not high-power LEDs. Thus, different cooling systems, such as the integrated multifin heat sink design with a fan, cooling systems featuring a thermoelectric cooler (TEC), and heat pipe cooling devices, are used to cool high-power LEDs [1, 4, 12]. High-power LEDs and LED devices are manufactured using different materials and operated at different power levels and in different configurations. The success of the thermal management of such LED devices varies with the cooling systems employed. In other words, an effective cooling system in a certain LED configuration may not be effective if there is a change in the LED configuration. To conclude, each parameter of LED devices and cooling systems should be evaluated simultaneously to choose a cooling system that is appropriate for the thermal management of LEDs [4, 14].

To the knowledge of the author although considerable researches have been performed regarding LEDs cooling systems in the available literature, different cooling systems used for high-power LEDs and comparisons in this study were not studied previously. Therefore, this study aimed to determine suitable cooling systems for high-power multichip LEDs. To this end, the effect of the dimidiation of LED power on temperature values, two different LED configurations, 30 W, and series-connected 2 × 15 W were used.

## 2. Theoretical Analysis

**Figure 1:**
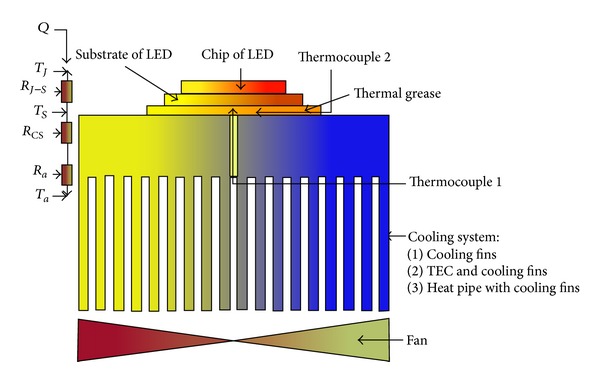
Schematic diagram of the LED system and thermal equivalent circuit of the heat transfer mechanism.

Approximately 80% of the electrical energy used in high-power LED devices is converted to heat [4]. A part of this heat is dissipated to the environment from the lighting and lateral surfaces of an LED by natural convection and radiation as a result of the temperature differences between the LED and the environment. However, the amount of heat transferred from these surfaces is small compared to the total amount of heat that should be extracted from the LED device. Consequently, the LED chip, where the maximum temperature occurs, transfers heat to the substrate of the LED, then to the thermal paste that is used to reduce thermal contact resistance and finally to the cooling system by heat conduction. As an additional fan is used in cooling systems, the next heat transmission process occurs from the cooling fins to ambient air through forced convection. An equivalent thermal circuit of this heat transfer mechanism and a schematic figure of an LED device are shown in Figure 1. The heat transferred between the junction of the LED chip and the substrate of LED is equal to that transferred between the junction of the LED and ambient air and can be defined as follows:

(1) $$\begin{array}{c} Q=\frac{\left(T_{J}-T_{S}\right)}{R_{J-S}}=\frac{\left(T_{J}-T_{a}\right)}{R_{t}}, \end{array}$$

where *Q*, *T*_*J*_, *T*_*S*_, *R*_*J*−*S*_, *T*_*a*_, and *R*_*t*_ refer to heat transmission, the temperature of the LED junction, the temperature of the substrate of LED, the thermal resistance from the LED junction to the substrate of LED, the environmental temperature, and the total thermal resistance, respectively. Thermal resistance is defined as follows:

(2) $$\begin{array}{c} R_{t}=R_{J-S}+R_{\text{CS}}+R_{a}, \end{array}$$

where *R*_*J*−*S*_, *R*_CS_, and *R*_*a*_ refer to the thermal resistance from the LED junction to the substrate of LED, the thermal resistance of the cooling system, and the thermal resistance of ambient air, respectively [4, 15]. As the junction of the LED chip is an internal component of the device, the junction temperature (*T*_*J*_) cannot be measured directly. In (1), *T*_*J*_ can be calculated by using (3):

(3) $$\begin{array}{c} T_{J}=T_{S}+Q\left(P\times 80\%\right)\times R_{J-S}. \end{array}$$

In (3), the heat transmission is equal to 80% of the LED input power. The thermal resistance from the LED junction to the aluminum substrate of the LED is defined as 0.2–0.5°C/W by the manufacturer of the device [12]. Thus, given the temperature of the substrate of the LED, the temperature of the LED junction can be determined because the other parameters in (1) are known [4, 12]. According to (1), it is clear that heat transmission changes in direct proportion to the difference between the temperature of the LED junction and the ambient temperature and in inverse proportion to the total heat resistance. It is not possible to control the temperature of the LED junction specific to LED manufacturing or the change in the ambient temperature of the environment where the LED is used by determining the amount of heat dissipated from the LED device efficiently by means of the temperature difference. Because of this restriction, the efficient extraction of heat from the LED device is only possible by developing high-performance cooling systems and materials for reducing the total heat resistance. The high temperature of the LED junction adversely affects the LED's life span, light quality, and power consumption, and thus the temperature should be maintained within a certain limit. If heat control for an LED device is provided by an effective cooling system, the temperature of the LED junction should remain within this limit [4, 12].

## 3. Experimental

### 3.1. Experimental System

**Figure 2:**
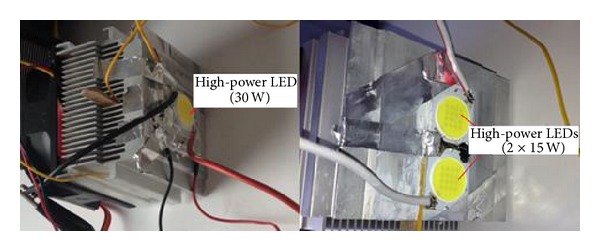
High-power multichip LEDs.

**Figure 3:**
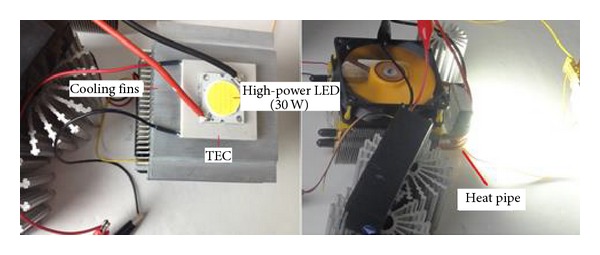
Cooling system with thermoelectric cooler (TEC) and heat pipe cooling device.

**Figure 4:**
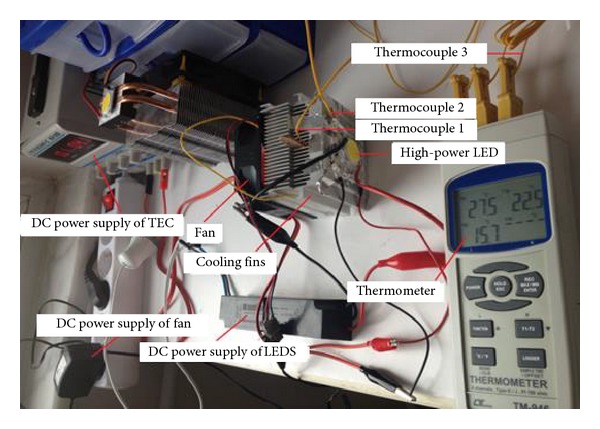
Experimental setup and cooling system (an integrated multifin heat sink design with a fan).

In the experimental systems used for this study, a Bridgelux brand multichip high-power LED was used in two different configurations, shown in Figure 2. The dimensions of the substrate of the LED were 22 × 25 mm. The LED models and power used were the BXRA-56C2600-H-00 model and the 30 W and BXRA-C1202 model serially connected to operate at 15 W [11]. To cool the LEDs, the cooling systems presented in Figures 3 and 4 were used: an integrated multifin heat sink design with 80 × 45 × 85 mm of aluminium fin a Cooler Master model 80 × 80 × 25 mm of fan supplying 30 CFM flow rate at 2200 RPM; a thermoelectric cooling unit obtained by integrating a TEC1-12708 model thermoelectric element, operating at a maximum temperature of 68°C with dimensions of 40 × 40 mm, into the same heat sink design [16]; and a heat pipe cooling device with Cooler Master RR-T2MN-22fp-R1 model 74 × 45 × 88 mm of aluminium fin and 80 × 80 × 25 mm of fan supplying 30 CFM flow rate at 2200 RPM and with two copper pipes [17].

**Figure 5:**
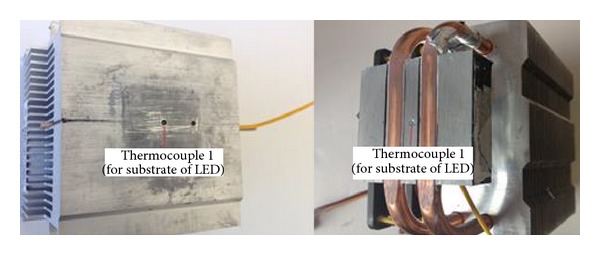
Thermocouple 1 for used temperature substrate of LED.

The temperature was measured with a 4-channel, Lutron brand TM-946 model thermometer. To these channels, K-type thermocouples with a measurement range of −199.9°C to 1370°C and a sensitivity of 0.1°C were connected [18]. Thermocouple 1, connected to the thermometer as shown in Figure 4, was used to measure the temperature of the substrate of the LED and TEC hot surface. Thermocouple 2 was used to measure the top surface temperature of the cooling system, and thermocouple 3 was used to measure the ambient temperature. As shown in Figure 5, channels were opened to surface cooling fins used in experimental study. Then full contact was provided for the surface of the substrate of the LED and TEC hot by passing the thermocouple 1 from these channels to sensitively measure the temperature of the substrate of the LED and TEC hot surface. Moreover, thermal paste was applied between the LED substrate and the top surface of the cooling unit to prevent contact thermal resistance. The experiment system was prepared with the junction of the system elements.

### 3.2. Application of the Experiment

LEDs systems that have active cooling systems reach heat balance in 5–10 minutes [4, 5, 12]. The LED or LEDs and the cooling fan used in each system were powered at the same time. The experiment involving the thermoelectric element was begun by powering the thermoelectric element. The duration of the experiment was monitored using a chronometer, and temperature values were recorded at certain time intervals. The experiment was conducted until the temperature reached a stable value. In the experimental study it was observed that measured temperature of samples becomes constant after 2–5 minutes from the beginning of experimental study. Measurements were performed for 12 minutes. In addition, the experiment was not begun until the temperature reached the ambient temperature. During the experiment, the ambient temperature was approximately 22.5°C.

## 4. Experimental Results and Discussion

**Figure 6:**
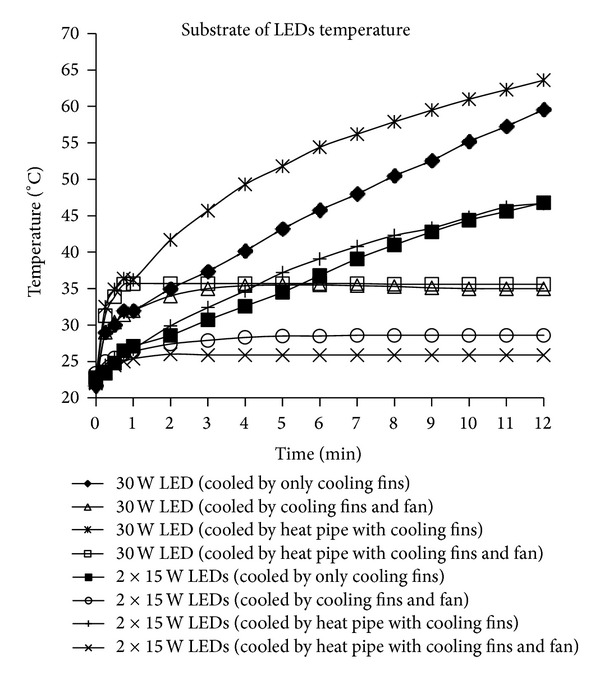
The change of the substrate of LED temperature over time during the cooling process of LEDs with 30 W and 2 × 15 W LEDs configuration as a means of integrated fin heat sink design with or without fan and heat pipe cooling device.

The temperature of the LED substrate was determined to be 108°C by substituting the numerical values used in the experimental LED systems into (3), where the maximum temperature of the LED junction was 120°C, the LED input power was 30 W power, and the thermal resistance from the LED junction to the substrate of the LED was 0.5°C/W. To create a durable, effective, and reliable LED configuration, the cooling systems used in the LED system must be maintained at a temperature below that calculated for the LED. The change in the LED substrate temperature over time during the cooling of the LEDs with the 30 W and 2 × 15 W LEDs configuration using the integrated fin heat sink design with or without a fan and a heat pipe cooling device is presented in Figure 6. According to Figure 6, when the fan was not used in both systems, the temperature increased at a constant rate after the ambient temperature was reached. As shown in Figure 6, during the 12th minute of the experiment, the fan was not run and the temperature of the substrate of LED of the 30 W LED decreased for both cooling devices reaching a temperature of approximately 60°C. When the fan was running, the temperature of the substrate of LED was 35°C for the 30 W LED. To prevent an increase in temperature, the cooling systems capacity should be enhanced or a fan should be integrated into the same system. In Figure 6, the temperature of the substrate of LED in three cooling systems used in the experiments is seen to be low compared to passive cooling systems. Low temperature of the substrate of LED is kept with decreasing of total thermal resistance due to the use of active cooling system in cooling LEDs system [1, 4].

**Figure 7:**
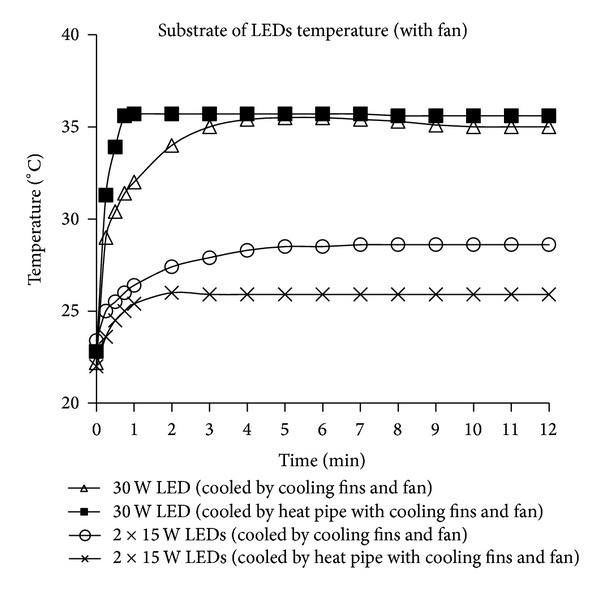
The change of the substrate of LED temperature overtime during the cooling process of LEDs with 30 W and 2 × 15 W LEDs configuration as a means of integrated fin heat sink design with fan and heat pipe cooling device.

The change in the LED substrate temperature over time during the cooling process of the LEDs adopting the 30 W and 2 × 15 W LED configurations using an integrated fin heat sink design with a fan and a heat pipe cooling device is presented in Figure 7. Whereas the substrate temperature of the 30 W LED was approximately 35°C in both cooling systems, this value was 26°C when the heat pipe cooling device was used for the 2 × 15 W LEDs. This result indicates that the amount of heat generated by the 2 × 15 W LEDs system was less than that generated by the 30 W LED device and that the heat extraction capacity of the heat pipe cooling device was more efficient during the cooling process of the 2 × 15 W LEDs system.

**Figure 8:**
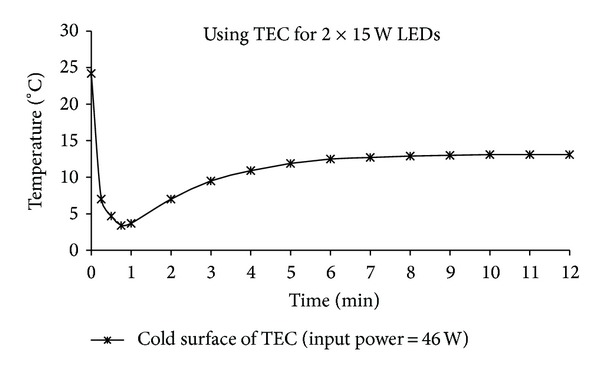
The change of the cold surface temperature of the TEC in the 2 × 15 W LEDs system.

For the thermoelectricity cooling unit used to cool both types of LEDs, three different powers were applied to the TEC (2.62 A × 10 V = 26 W, 3.3 A × 13.9 V = 46 W, and 3.6 A × 15 V = 54 W) and the hot and cold surface temperatures were measured. The change in the cold surface temperature of the TEC in the 2 × 15 W LEDs system over time is presented in Figure 8. As shown in Figure 8, the temperature, which was initially equal to the ambient temperature, fell dramatically to 3°C within 45 seconds, then increased to 13°C in the seventh minute and remained stable at this value. This behaviour is a result of the faster cooling effect of the TEC element when initially compared to the heat generated by the LEDs. Over time, the heat generated by the LEDs passed to the substrate of the LED, TEC element, cooling fin, and ambient air through the fan and a balance in temperature was obtained. It is clear that cooling systems in which TEC elements are used exhibit a rapid response.

**Figure 9:**
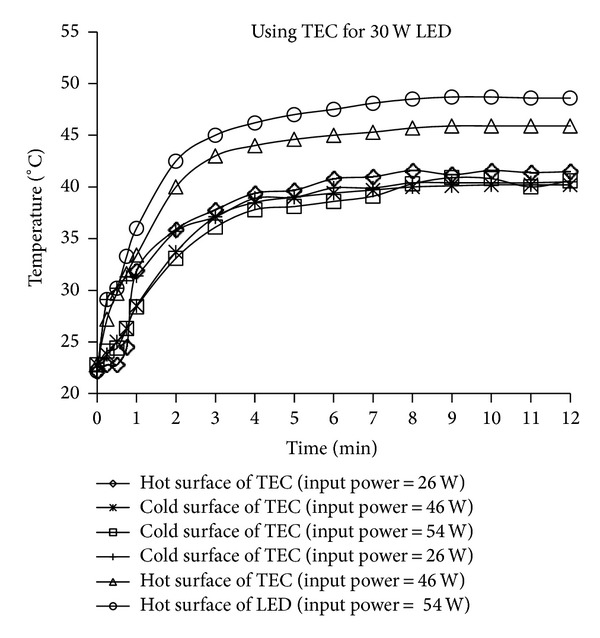
The cold and hot surface temperature values of the TEC element during the cooling process of the LEDs with the 30 W LED configuration via cooling systems in which the TEC elements were used.

**Figure 10:**
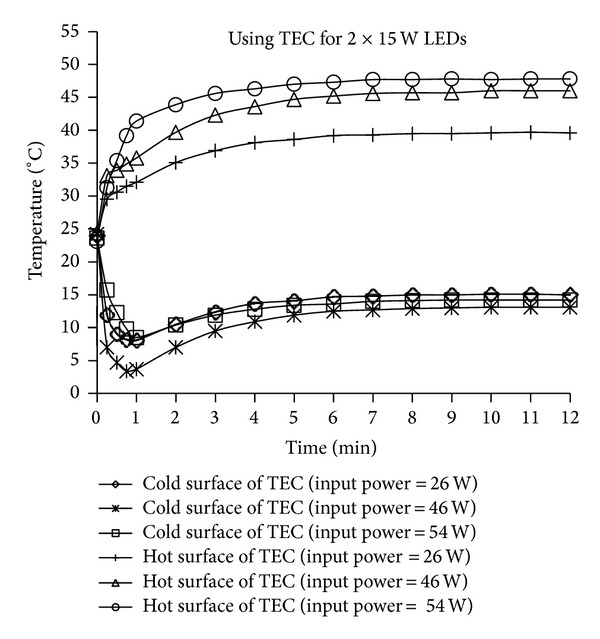
The cold and hot surface temperature values of the TEC element during the cooling process of the LEDs the 2 × 15 W LEDs configuration via cooling systems in which the TEC elements were used.

The cold and hot surface temperature values of the TEC element during the cooling process of the LEDs with adopting 30 W and the 2 × 15 W LEDs configurations via cooling systems in which the TEC elements were used are presented in Figures 9 and 10.  Figure 9 shows that the hot and cold surface temperatures for the three different powers applied to the TEC of the 30 W LED are similar. The lowest temperature for the 30 W LED was observed when a power of 54 W was applied to the TEC. By comparing this power applied to the TEC with that applied to the passive cooling systems in which the fan was not used (Figure 6), it is clear that the temperature of the LED device can be controlled. However, the cooling TEC element is not sufficient to fully cool the device, and thus higher-capacity TEC elements are needed.

According to Figure 10, there is a difference between the hot and cold surface temperatures of the TEC among the three different powers applied to the TEC in the 2 × 15 W LEDs device, and it is clear that the cooling process was effective at all three power values. The lowest temperature was obtained when 46 W of power was applied to the TEC, with the temperature ranging from 13 to 14°C among the three power values.

**Figure 11:**
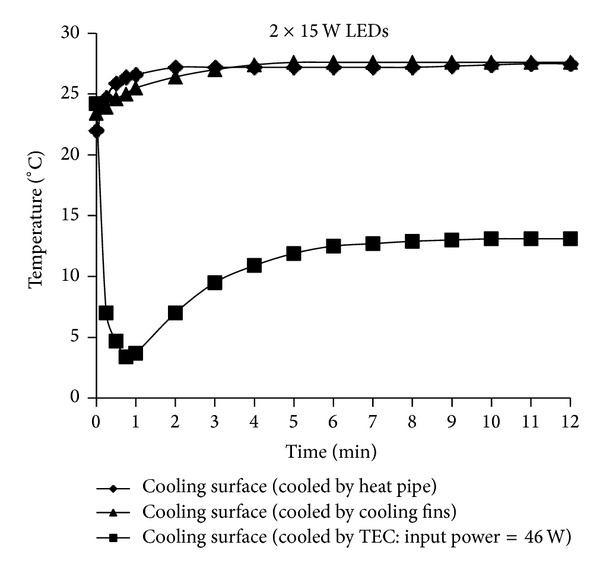
The temperature-time graphics of three different cooling systems used to cool LEDs with 2 × 15 W LEDs configuration.

**Figure 12:**
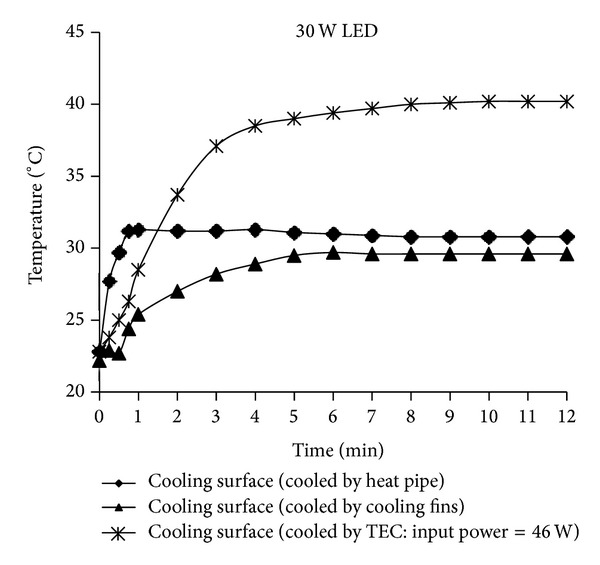
The temperature-time graphics of three different cooling systems used to cool LEDs with 30 W LED configuration.

The temperature-time curves of the three different cooling systems used to cool the LED devices adopting the 30 W and 2 × 15 W LEDs configurations are presented in Figures 11 and 12. According to Figure 11, each of the three systems was sufficient for cooling the 2 × 15 W LEDs and the power applied to the TEC in the best cooling system with a TEC element was 46 W. The other two cooling systems showed approximately the same cooling effect. According to Figure 12, each of the three systems was sufficient for cooling the 30 W LED and the best cooling was obtained with an integrated fin heat sink design. This performance was followed by that of the heat pipe cooling device and cooling system with a TEC element.

The experiments showed that the use of passive cooling system is not enough in the cooling of high-power LEDs, the temperature of the substrate of LED is constantly high, and thermal control of high-power LED system is not kept. In order to keep the thermal control of high-power LEDs, it is imperative to use constantly active cooling systems.

As the fans used in active cooling system consume about 2-3 W of power, their effect on cooling efficiency can be disregarded due to their benefits. In cooling system with TEC, as power applied on the TEC is similar to the power that LED uses, cost-benefit analysis should be done.

## 5. Conclusions

The results obtained in this study show that an integrated fin heat sink design with a fan, heat pipe cooling device, and cooling system with a TEC element are sufficient to cool LEDs with a 30 W or 2 × 15 W LEDs configuration. Results showed that the temperature capacity of the cooling system with a TEC element is the most efficient for the 30 W LED when 54 W power applied to TEC. At the same time, compared to other cooling systems is the most inefficient. Obtained results indicated that, cooling system with a TEC element is the most efficient for the 2 × 15 W LEDs device. The used power of TEC element should be considered if in the cooling system with a TEC element is preferred for the cooling of 30 W and more power LEDs. If passive (without fan) cooling systems are used to cool high-power LEDs, their volumes and capacities should be greatly enhanced. Such an enhancement, however, would lead to some difficulties such as large occupied volume, difficulties associated with mounting, and poor aesthetic appearance. Thus, active cooling systems are preferred in such LED devices. Further studies are also needed to investigate the cost analyses for the mentioned different three cooling systems.

## Nomenclature

*P*: Input power, W
*Q*: Heat transfer, W
*R*: Thermal resistance, °C/W
*T*: Temperature, °C.

### Subscripts

*a*: Ambient
cs: Cooling system
*J*: Junction
*J* − *s*: From the LED junction to the substrate of LED
*s*: Substrate
*t*: Thermal.
